# Unperceived motor actions of the balance system interfere with the causal attribution of self-motion

**DOI:** 10.1093/pnasnexus/pgac174

**Published:** 2022-08-27

**Authors:** Romain Tisserand, Brandon G Rasman, Nina Omerovic, Ryan M Peters, Patrick A Forbes, Jean-Sébastien Blouin

**Affiliations:** School of Kinesiology, University of British Columbia, Vancouver, BC V6T 1Z1, Canada; Institut PPRIME (UPR3346), Université de Poitiers ENSMA, CNRS, 86360 Chasseneuil-du-Poitou, France; Centre de Recherches sur la Cognition et l'Apprentissage (UMR 7295), Université de Poitiers, Université de Tours, CNRS, 86073 Poitiers, France; Department of Neuroscience, Erasmus MC, University Medical Center Rotterdam, Rotterdam 3015 GD, The Netherlands; School of Physical Education, Sport, and Exercise Sciences, University of Otago, Dunedin 9054, New Zealand; Department of Neuroscience, Erasmus MC, University Medical Center Rotterdam, Rotterdam 3015 GD, The Netherlands; School of Kinesiology, University of British Columbia, Vancouver, BC V6T 1Z1, Canada; Faculty of Kinesiology, University of Calgary, Calgary, AB T2N 1N4, Canada; Department of Neuroscience, Erasmus MC, University Medical Center Rotterdam, Rotterdam 3015 GD, The Netherlands; Department of Biomechanical Engineering, Delft University of Technology, Delft, The Netherlands; School of Kinesiology, University of British Columbia, Vancouver, BC V6T 1Z1, Canada; Institute for Computing, Information & Cognitive Systems, University of British Columbia, Vancouver, BC V6T 1Z4, Canada; Djavad Mowafaghian Center for Brain Health, University of British Columbia, Vancouver, BC V6T 1Z3, Canada

**Keywords:** perception, action, standing, postural sway, balance control, psychophysics, perturbation

## Abstract

The instability of human bipedalism demands that the brain accurately senses balancing self-motion and determines whether movements originate from self-generated actions or external disturbances. Here, we challenge the longstanding notion that this process relies on a single representation of the body and world to accurately perceive postural orientation and organize motor responses to control balance self-motion. Instead, we find that the conscious sense of balance can be distorted by the corrective control of upright standing. Using psychophysics, we quantified thresholds to imposed perturbations and balance responses evoking cues of self-motion that are (in)distinguishable from corrective balance actions. When standing immobile, participants clearly perceived imposed perturbations. Conversely, when freely balancing, participants often misattributed their own corrective responses as imposed motion because their balance system had detected, integrated, and responded to the perturbation in the absence of conscious perception. Importantly, this only occurred for perturbations encoded ambiguously with balance-correcting responses and that remained below the natural variability of ongoing balancing oscillations. These findings reveal that our balance system operates on its own sensorimotor principles that can interfere with causal attribution of our actions, and that our conscious sense of balance depends critically on the source and statistics of induced and self-generated motion cues.

Significance statement As we move throughout the world, the brain must dissociate between self-generated motion and external disturbances to maintain accurate perception and control of movements. While standing, this process is used to extract, identify and respond to external stimuli threatening stability. Here, we demonstrate a paradox in human standing, where actions of the balance motor system can be perceived as external disturbances because they are generated outside of perceptual awareness. Critically, this occurs only when perturbations are encoded ambiguously within the natural variability of the ongoing balancing behavior. We conclude that the balance motor system operates on its own principles that elude perception and can impede causal attributions of self-motion.

## Introduction

To maintain accurate perception and control of our movements, the brain integrates sensory information to build internal representations of the body and world. During most daily activities, the actions of the motor system facilitate this process because the brain uses the knowledge of planned and executed motor commands to determine whether movements arise from self-generated actions or external disturbances (1–3). When standing upright, this distinction is critical for detecting imposed whole-body motion embedded within self-generated postural oscillations in order to produce corrective balance responses only to external disturbances that may threaten stability. A longstanding theory assumes that a single internal representation of the body and world is relied upon for both the accurate sense of postural orientation and the organization of motor responses that control balance (4–7). However, this view is at odds with the observation that some motor actions of the balance system elude our conscious perception (8–11), suggesting that our ability to sense and control balance do not both arise from a single representation (11, 12). As a result, we propose that the postural actions produced by the balance motor system may interfere with the causal attribution of perceived balance self-motion. Here, we establish how the corrective control of upright standing can distort the human sense of balance.

The sense of balance can be quantified with perceptual thresholds to imposed whole-body and/or lower limb perturbations (13–18). However, the majority of studies (13–15, 18) examining these perceptions used immobilization devices that removed the balance system’s contribution to upright self-motion due to the body being braced (10, 19, 20). Highlighting the importance of characterizing the perceptual processes while participants balance, Teasdale et al. (16) observed that participants reported self-generated whole-body motion as imposed motion during catch trials; i.e. trials without imposed motion. Critically, these authors also reported that participants moved in the direction opposite to the imposed motion even in trials that were unperceived. Perceptions of self-generated balancing movement as an imposed perturbation were also observed when transmastoid electrical stimuli was used to modulate vestibular afferent activity (21, 22). Wardman et al. (23) reported that participants perceived the expected direction of the vestibular-induced virtual motion (24–26) when braced upright to a backboard; but, when free to balance, participants only perceived their whole-body motion in the direction of the evoked balance response (as opposed to the direction of the vestibular perturbation). Together, these results stress the need to characterize the sense of balance when our balance system is engaged and generates corrective responses. They further suggest that, although the direction of an imposed perturbation may not be consciously perceived when balancing upright, our balance control system can detect small imposed perturbations, identify them within ongoing postural oscillations, and elicit a balance response in the opposite direction that may, in turn, give rise to a conscious perception of self-motion.

Another important consideration for the conscious perception of standing balance relates to how the contribution of specific sensory cues can lead to distinct contexts for perceiving self-motion (13, 14). When perturbations are delivered to the whole-body, self-motion cues are detected by somatosensory, vestibular and visual sensors, first in the direction of the imposed perturbation and then in the direction of the balance response. Because the same sensory cues of self-motion encode whole-body movement in both directions and participants are asked to report their whole-body motion, ambiguous perceptions of the imposed motion can arise. In particular, perturbations that are smaller than the natural whole-body oscillations while standing (i.e. natural statistics of balance) may evoke balance responses without participants perceiving the induced motion (8). As a key feature of this ambiguity, we propose that the conscious perception of balance to whole-body perturbations depends on the relative magnitude of the externally imposed versus balance-generated self-motion. Support surface perturbations (i.e. rotation of the ankle joints) are also likely to evoke whole-body balance responses when the control of standing balance is engaged. However, unlike whole-body perturbations, ankle rotations target specifically feet and ankle somatosensory cues, such that the resulting whole-body balance correcting responses are encoded by multiple sensory sources, including vestibular and visual cues. As a result, it may be possible that participants asked to detect ankle motion may disambiguate the sensory coding of ankle motion resulting from perturbations and balance responses because only the balance correcting responses involve vestibular and visual cues related to whole-body movements.

Here, we aimed to reveal the principles underlying our sense of standing balance by quantifying perceptual thresholds to perturbations inducing context-dependent ambiguous or unambiguous cues of self-motion. Healthy participants stood immobile or balanced freely on a robotic balance simulator (27–30) while their perception thresholds to imposed whole-body or ankle perturbations were measured across a range of perturbation velocities. We estimated perception thresholds to the imposed perturbations by fitting the data with psychometric curves. When standing upright but immobile, we hypothesized that participants would perceive only the direction of the imposed (whole-body and ankle) perturbations, and therefore exhibit a single threshold to the perturbation. Due to the ambiguous cues of self-motion resulting from whole-body perturbations applied when balancing upright, we then hypothesized that participants instructed to focus on their whole-body motion would perceive the direction of their self-generated balance response for small perturbation velocities because they remain unaware of some of the actions of their balance control system (8–10, 16). For larger whole-body perturbations applied while balancing, we predicted participants would perceive the direction of the imposed whole-body motion. Hence, we expected participants would exhibit two distinct perceptual thresholds: a lower threshold in the direction opposite to the imposed perturbation and a higher threshold in the direction of the imposed perturbation. To explain how participants were more likely to report the direction of their balance response or the direction of the perturbation as the perturbation velocity increased, we developed a model combining two psychometric functions with a weight attributed to cues related to whole-body motion in each direction that varied as a function of the imposed perturbation velocity. Finally, we hypothesized that freely balancing participants asked to focus on their ankle movements and exposed to ankle perturbations would perceive only the direction of the imposed perturbation (i.e. a single perceptual threshold) even in the presence of a corrective balance response because the imposed ankle motion can be disambiguated from the whole-body balance response.

## Results

### Perception of whole-body motion: ambiguous cues of whole-body movement lead to perceptions of self-generated balance responses as imposed motion

**Fig. 1.**
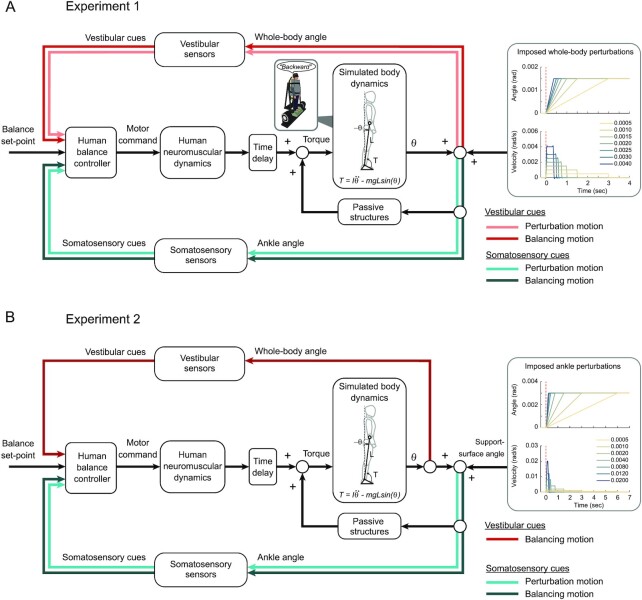
Control loop of the robotic balance simulator used to mimic and perturb standing balance. For both Experiment 1 (A) and Experiment 2 (B), participants were standing on the robotic balance simulator (thick gray panel), reproducing the whole-body balance dynamics in the sagittal plane. The differential equation used to simulate the dynamics of standing balance is based on an inverted pendulum model with the following parameters: *L* = height from ankles to center of mass (open circle), *θ* = angular displacement of the center of mass from vertical (dashed line: a clockwise angular displacement is negative and a counter-clockwise displacement is positive), *T* = torque (a counter-clockwise torque is positive whereas a clockwise torque is negative), *I* = mass moment of inertia of the whole-body, *m* = whole-body mass, and *g* = gravitational constant. Torque *T* was directly measured from the force plate data (green square underneath participants’ feet) to estimate the whole-body angle *θ*. Vestibular and somatosensory cues arising from whole-body and ankle angle inputs are depicted on separate feedback loops for each sensory cue. Sensory cues arising in the direction of the perturbation are depicted with light colored lines while sensory cues arising in the direction of the balance response are depicted as dark colored lines. Passive structures (stiffness and damping) and time delays (capturing sensory transduction, neural processing, transmission, and muscle activation delays) are depicted for completeness. (A) In Experiment 1, imposed whole-body perturbations were added to the computed *θ* within the control loop and modified both the whole-body and ankle angles. For clarity, the characteristics (angle and velocity) of only the seven largest perturbations (from 0.0005 to 0.0040 rad/s) are depicted. Somatosensory and vestibular cues encode both perturbation and balancing motion. (B) In Experiment 2, imposed ankle perturbations were added to the computed *θ* within the control loop but modified only the ankle angle through support-surface rotations. Vestibular cues (light red in Experiment 1) are not explicitly targeted by the perturbation motion and are absent when ankle motion is imposed. For both experiments, participants were exposed to perturbations and asked to verbally report the direction(s) in which they perceived a whole-body (i.e. forward/backward) or ankle (toes-up/toes-down) motion was imposed. Verbal report was binary encoded (1 for a correct response, 0 for an incorrect response).

To determine whether and how imposed whole-body perturbations and balance generated self-motion interact to induce ambiguity in the conscious perception of standing balance, in Experiment 1, we applied whole-body perturbations in the anterior–posterior plane to standing participants (*N* = 10) at nine velocities (range: 0.0001 to 0.0040 rad/s) with a fixed displacement of 0.0015 rad (Fig. 1A). Participants stood quietly on a robotic balance simulator (see the “Materials and methods” section) while immobile or balancing in the anterior–posterior plane and reported on the perceived direction (i.e. forward and/or backward) of the imposed whole-body perturbations. To isolate sensory feedback of whole-body motion to vestibular and somatosensory cues, participants wore a blindfold, earplugs, and noise canceling headphones to minimize visual and auditory cues associated with movements of the robot.

#### Whole-body motion to imposed whole-body perturbations

We first quantified the presence and magnitude of the perturbation-evoked balance responses to determine whether these whole-body movements were larger than either the imposed perturbation or the natural oscillations (i.e. statistics) of standing prior to the perturbations. For the Immobile condition, the perturbation moved the participants’ whole-body 0.0015 rad only in the direction of the perturbation, and then remained stationary until the end of the trial (Fig. S1A). For the Balancing condition, participants stood upright with small oscillations in whole-body sway: the variability (SDs) of whole-body position and velocity during the 1.5 s preceding the delivery of the perturbation were 0.0012 ± 0.0001 rad and 0.0022 ± 0.0006 rad/s, respectively. The applied perturbations moved the participants’ whole-body in the direction of the perturbation, and this movement was followed by a detectable balance response (i.e. >2 SDs of whole-body position preceding perturbation) in the opposite direction in most of the trials (54% ± 11 and 70% ± 8 of the time, respectively for the 0.0001 and 0.00025 rad/s perturbation velocities; 100% of the time for all other perturbations velocities, Fig. S1B). Notably, the 70% detection of balance responses at the 0.00025 rad/s perturbation reflects a velocity threshold for evoking a balance response. Above this perturbation threshold, participant-averaged peak angular displacements and velocities in the direction of the balance response across all perturbations ranged from ∼0.0119 to 0.0153 rad and ∼0.0086 to 0.0117 rad/s, respectively. Participants were also never under any visible threat to balance as a result of the perturbations and they never fell into the virtual limits of the balance simulation (i.e. 0.10 rad anterior and 0.05 rad posterior). These results show that whole-body perturbations > 0.00025 rad/s elicited a consistent balance response with peak angular displacements and velocities that were ∼3 to 17 times larger than the imposed perturbations and ∼4 to 12 times larger than 1 SD of the oscillations of natural sway preceding the perturbation.

#### Perception performance and thresholds to whole-body perturbations

**Fig. 2.**
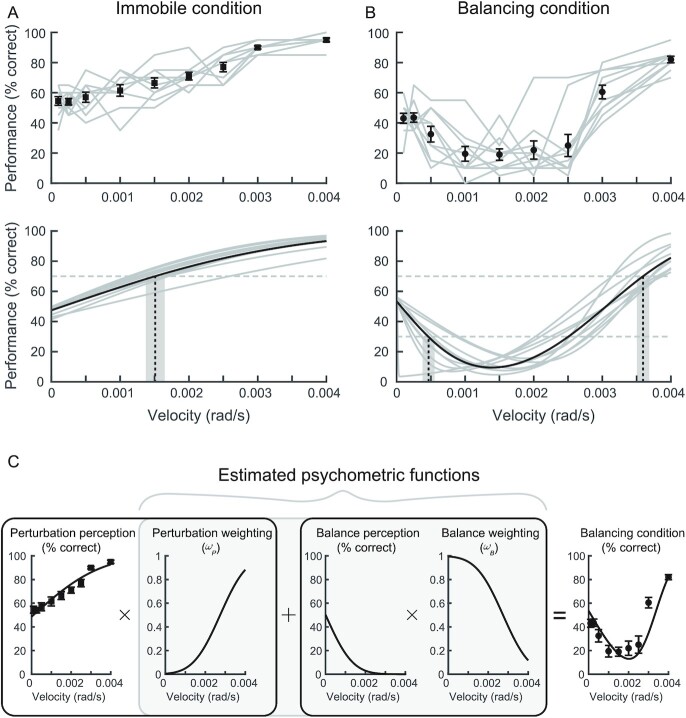
Perception performance and thresholds to whole-body perturbations (Experiment 1). (A) Perception performance (top) and fitted psychometric functions (bottom) for the Immobile condition. Perception performance in the Immobile condition was best fit with a single psychometric function. (B) Perception performance (top) and fitted psychometric functions (bottom) for the Balancing condition. Perception performance in the Balancing condition was best fit with a dual psychometric function. Gray lines in both (A) and (B) are individual participants and the black lines/symbols (and error bars) are the group averages (and SEMs). Dashed vertical lines and grayed regions in the psychometric functions are the group average thresholds and accompanying SEMs, respectively (Immobile: 0.0016 ± 0.0004 rad/s [70% threshold]; Balancing: 0.00045 ± 0.0003 rad/s [30% threshold] and 0.0036 ± 0.0003 rad/s [70% threshold]). Horizontal dashed lines are the 30% and 70% limits used to identify the thresholds. (C) A weighted model of sensorimotor integration explains the perception of whole-body perturbations when balancing. The probability of perceiving the direction of the perturbation (first panel; *μ* = 0.00009 rad/s and *σ* = 0.00269 rad/s) and perceiving the direction of the balance response (third panel; *μ* = 0.00001 rad/s and *σ* = 0.00106 rad/s) were multiplied by their respective estimated weighting functions (*ω_P_* and *ω_B_*, second and fourth panel; both *μ* = 0.00275 rad/s and *σ* = 0.00106 rad/s) and then summed together to predict the perception performance in the Balancing condition (fifth panel). Note, the perception data from the Immobile condition (A) were used to model the probability of perceiving the direction of the perturbation (left panel in C). The perception data from the Balancing condition (B) were then used to estimate psychometric functions for the probability of perceiving the direction of the balance response (i.e. Balance perception) and the accompanying weights (*ω_P_* and *ω_B_*), as highlighted in the shaded gray region (“Estimated psychometric functions”).

We next evaluated the participants’ rate of correct perception to imposed whole-body perturbations to test whether the direction perceived depends on the magnitude of the externally imposed motion. In the Immobile condition, participants correctly perceived the perturbation direction at chance level for small velocities (54 ± 9% and 54 ± 7% for 0.0001 and 0.00025 rad/s, respectively). The rate of correct perception then progressively increased as the perturbation velocity increased, reaching a maximum of 95 ± 4% for the largest velocity (Fig. 2A). In the Balancing condition, participants also exhibited a rate of correct perception close to 50% for the two smallest velocities (43 ± 10% and 44 ± 9% for 0.0001 and 0.00025 rad/s, respectively). But, as the perturbation velocity increased, the rate of correct detection first decreased, reaching a minimum of 19 ± 11% at 0.0015 rad/s, and then increased to reach a maximum of 82 ± 6% at 0.004 rad/s (Fig. 2B). These results suggest that participants balancing freely misattributed the direction of their balance responses as imposed motion for low velocity whole-body perturbations (0.001 to 0.0025 rad/s) but correctly perceived the direction of the perturbation for larger whole-body perturbations (>0.003 rad/s). Notably, the transition at the 50% correct level between perceiving the direction of the balance response and perceiving the direction of the imposed motion (∼0.0027 rad/s; see Fig. 2B) approximately aligned with the SD of whole-body sway velocity preceding the perturbation (0.0022 ± 0.0006 rad/s).

To describe the perception performance in each condition, we next computed two distinct curve fits (a single and a dual psychometric curve) for each participant in each condition (Immobile and Balancing; see the “Materials and methods” section and Supplementary Material Appendix for more details). The dual psychometric function accounted for the probability of perception in both the direction opposite to the perturbation (at low velocities) and the direction of the perturbation (at high velocities) observed during the Balancing condition. We assessed the quality of the two fits to capture the perception performance using the Akaike and Bayesian information criteria (AIC and BIC; see the “Materials and methods” section). We found that a single psychometric function was better suited (i.e. lower AIC/BIC; see Supplementary Material Appendix Table S1) to quantify the participants’ perceptual thresholds in the direction of the perturbation for the Immobile condition (observed in 9 of 10 participants, see Fig. 2A), while a dual psychometric function was better suited to quantify both the direction opposite to the perturbation and the direction of the perturbation for the Balancing condition (observed in 9 of 10 participants, see Fig. 2B). As a result, single psychometric functions and dual psychometric functions were subsequently used to quantify the participants’ perceptual thresholds in the Immobile and Balancing conditions, respectively.

Fitting the single psychometric curve to the Immobile condition showed that perception performance for all participants (*N* = 10) increased with perturbation velocity. This resulted in a perception threshold at 0.0016 ± 0.0004 rad/s (Fig. 2A), which decreased to 0.0015 ± 0.0004 rad/s when we removed the data from one participant that were better fit using a dual psychometric function. In the Balancing condition, the dual psychometric curve fitting for all participants (*N* = 10) started near 50%, but as the perturbation velocity increased, all curves initially decreased to a minimum from 4% to 15% and then progressively increased to a maximum from 72% to 97% (Fig. 2B). Subsequently, a threshold for perception of the direction of the balance response (i.e. incorrect detection of perturbation direction) was defined as the smallest velocity where participants reached 30% of correct perception. This threshold for all participants was 0.00047 ± 0.00028 rad/s, which increased to 0.00051 ± 0.00025 rad/s when we removed the data from one participant that were better fit using a single psychometric function. The threshold for perceiving the direction of the perturbation (i.e. 70% correct detection of perturbation direction) was 0.0036 ± 0.0003 rad/s and did not change when the data from the one participant that were better fit by a single psychometric function were removed. These three perceptual thresholds differed (*F*_(9,2)_ = 225.8, *P* < 0.001), with the threshold for perceiving the incorrect direction of the balance response in the Balancing condition being on average 72% and 88% lower than the thresholds for perceiving the correct direction of the perturbation in the Immobile (*P* < 0.01) and the Balancing (*P* < 0.001) conditions, respectively. Also, the threshold for perceiving the direction of the perturbation in the presence of the natural oscillations of balance (i.e. the Balancing condition) was approximately two times higher than when participants were immobile (*P* < 0.001). These findings suggest that at low perturbation velocities, participants did not perceive the perturbation, were unaware of the corrective response generated by their balance system, and consequently perceived this balance response as the imposed motion.

We next reasoned that if a participant’s perception of the perturbation during balance is linked to the experienced motion (imposed and balance correction), then the angular displacement and velocity peaks would vary depending on whether they perceived the direction of the imposed perturbation (i.e. correct response) versus the opposite direction of the perturbation (i.e. incorrect response). In general, peak angular position and velocity were 3 to 16× larger in the direction of the balance response than in the direction of the imposed perturbation (Fig. S2 and see Supplementary Material Appendix Table S2). When participants correctly perceived the direction of the imposed perturbation, their peak angular displacements (0.0013 to 0.0054 rad versus 0.0003 to 0.0015 rad, all *P* < 0.001) and velocities (0.0017 to 0.0033 rad/s versus 0.0009 to 0.0019 rad/s, all *P* < 0.001) in the direction of the perturbation were larger than when they (incorrectly) perceived the direction of the balance response, for both directions of the perturbation. (Note: reported ranges are across perturbation velocities; see Supplementary Material Appendix Table S2.) In contrast, when they (incorrectly) perceived the direction of the balance response, the participants’ balance responses were larger (0.014 to 0.020 rad versus 0.006 to 0.014 rad, all *P* < 0.001) and faster (0.009 to 0.012 rad/s versus 0.007 to 0.010 rad/s, *P* < 0.01 only in the backward direction) than when they correctly perceived the direction of the perturbation. (Note: reported ranges are across perturbation velocities; see Supplementary Material Appendix Table S2.) Overall, these results indicate that when participants (incorrectly) perceived the direction opposite to the perturbation, they experienced smaller imposed whole-body motion and generated larger balance responses than when they (correctly) perceived the direction of the perturbation.

#### A physiological model for perceiving whole-body perturbations

Although correctly fitting the experimental data in the Balancing condition, the dual psychometric curve fit did not explain how participants weighted the successive sensory signals encoding their whole-body self-motion to the imposed movement and their balance response to the perturbation. To address these limitations, we proposed a weighted model combining two psychometric functions with respective weighting functions. The weighting functions represent the probability that participants attribute the accompanying sensory cues to whole-body motion either in the initial direction of the imposed perturbation (*ω_p_*) or the subsequent direction of the balance response (*ω_B_*) (see Equation S1 and further details in Supplementary Material Appendix). The best-estimated weighting functions *ω_P_* and *ω_B_* were computed as a cumulative distribution function and reversed cumulative distribution function, respectively, where *ω_B_* = 1–*ω_P_*. The *ω_P_* and *ω_B_* curves had a *σ* = 0.00106 rad/s and crossed the 50% level (i.e. *µ*) at 0.00275 rad/s (Fig. 2C). This indicates that when participants experienced whole-body motions from both an imposed perturbation and their self-generated balance response, they preferentially weighted the balance response motion compared to the motion of the imposed perturbation when perturbation velocities were near or below the natural variability of standing balance behavior (i.e. <0.00275 rad/s or ≤1 SD of sway velocity [0.0022 ± 0.0006 rad/s]), and the opposite for perturbations delivered at higher velocities.

### Perception of ankle motion: imposed ankle motion is unambiguously perceived during standing balance

Our results so far suggest that standing participants asked to perceive their whole-body motion are largely unaware of their self-generated corrective balance responses to low-velocity whole-body perturbations, and perceive these movements as imposed motion. Next, we investigated whether this misattribution in the perception of experienced standing balance self-motion can be resolved when participants asked to perceive an imposed ankle perturbation can use sensory cues (i.e. vestibular) that accompany only the whole-body balance responses. To test this hypothesis, we delivered plantar- and dorsi-flexion ankle rotations to standing participants (*N* = 10) at seven velocities (range: 0.0005 to 0.02 rad/s) with a fixed displacement of 0.003 rad (Fig. 1B). Participants stood immobile or while balancing in the anterior–posterior plane and reported their perceived direction (i.e. toes-up or toes-down) of the imposed ankle perturbations.

#### Whole-body motion to imposed ankle motion

Similar to Experiment 1, we first quantified the presence and magnitude of the balance responses to determine whether these whole-body movements were larger than either the imposed ankle perturbation or the balance oscillations prior to the perturbation. In the Immobile condition, the backboard remained stationary throughout the perturbation and the participant’s ankles moved only in the direction of the perturbation and remained stationary until the end of the trial (see Fig. S3A). In the Balancing condition, participants stood upright with whole-body oscillations (estimated during the 1.5 s preceding the ankle perturbation) that resulted in displacement and velocities SDs of 0.0007 ± 0.0001 rad and 0.0015 ± 0.0003 rad/s, respectively. The imposed ankle perturbations moved the participants’ feet in the direction of the perturbation while participants balanced upright, producing either ankle dorsiflexion or plantarflexion for toes-up and toes-down perturbations, respectively. This imposed ankle motion was followed by a whole-body balance response in most trials (>90% for each perturbation velocity) that occurred in the same direction as the ankle rotation, i.e. backwards during toes-up perturbations and forwards during toes-down perturbations (see Fig. S3B). As a result, although the whole-body balance response was in the same direction as the ankle motion, the resulting ankle rotation was opposite to the imposed perturbation, i.e. plantarflexion for toes-up (dorsiflexion) perturbations and in dorsiflexion for toes-down (plantarflexion) perturbations. Across the ankle perturbation velocities tested, peak whole-body displacement and velocity evoked by the ankle perturbations ranged between ∼0.005 to 0.01 rad and ∼0.006 to 0.011 rad/s, respectively. Overall, these results show that the peak angular position and velocity of the balance response to ankle perturbations were ∼1 to 12 times larger than the imposed ankle perturbation and ∼4 to 14 times larger than 1 SD of the oscillations of sway immediately before perturbation.

#### Perception performance and thresholds to imposed ankle motion

**Fig. 3.**
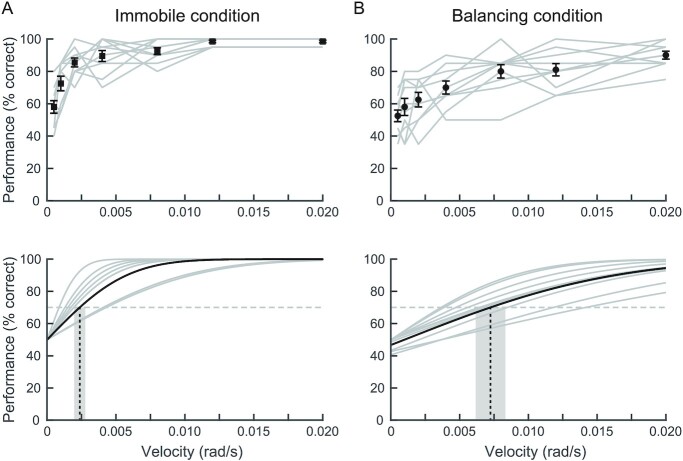
Perception performance and thresholds to ankle perturbations (Experiment 2). (A) Perception performance (top) and fitted psychometric functions (bottom) for the Immobile condition. (B) same as (A), but for the Balancing condition. Perception performance in both the Immobile and Balancing conditions were best fit with a single psychometric function. Gray lines in all plots are individual participants and the black lines/symbols (and error bars) are the group averages (and SEMs). Dashed vertical line and grayed regions in the psychometric functions are the group average thresholds and accompanying SEMs, respectively (Immobile: 0.0023 ± 0.0012 rad/s [70% threshold]; Balancing: 0.0073 ± 0.0034 rad/s [70% threshold]). Horizontal dashed lines are the 70% limits used to identify the thresholds.

We then evaluated the rate of correct perception to the imposed ankle perturbations to test whether participants would perceive only the direction of an imposed motion. In the Immobile condition, the rate of correct perception was close to chance (58% ± 12) at the smallest velocity of 0.0005 rad/s and progressively increased with perturbation velocity, approaching 100% (99 ± 2%) at the largest two velocities (see Fig. 3A). In the Balancing condition, a similar trend in the rate of correct perception was observed, though with nearly 50% of the trials detected at the two smallest velocities (53 ± 11% and 58 ± 2% for 0.0005 rad/s and 0.001 rad/s, respectively), which progressively increased to 90 ± 7% at the highest velocity (i.e. 0.020 rad/s). Notably, the average rate of change in perception performance during the Balancing condition occurred over a wider range of perturbation velocities as compared to the Immobile condition (see Fig. 3B). These results indicate that participants perceived only the direction of the ankle perturbation during both Immobile and Balancing conditions.

We next assessed the probability for a single or a dual psychometric curve fit to capture the observed perception performance and extracted the accompanying detection thresholds. We found that both the Immobile and Balancing conditions were better fit (lower AIC and BIC) by the single than the dual psychometric function (both observed in 9 of 10 participants, see Supplementary Material Appendix Table 1 and Fig. 3A and B). Fitting a single psychometric curve to each participant’s responses showed that perceptual performance for both Immobile and Balancing conditions started at ∼50% for the lowest velocity and increased to 90%–100% for the highest velocity for both conditions. The perceptual threshold estimated for the Immobile condition was 0.0023 ± 0.0012 rad/s, which increased to 0.0024 ± 0.0012 rad/s when we removed the data from the one participant that were better fit by a dual psychometric function. In contrast, the threshold for Balancing condition was approximately three times larger than the Immobile condition (0.0073 ± 0.0034 rad/s; paired *t*-tests: *t*_(18)_ = 4.3, *P* < 0.001). This threshold also slightly increased (0.0074 ± 0.0036 rad/s) when removing the data from the one participant that were better fit by a dual psychometric function. This suggests that when balancing upright, imposed ankle motions must be approximately three times larger than those experienced when standing immobile to correctly perceive imposed ankle rotations. Furthermore, although participants could not perceive the imposed movements at perturbation velocities below this threshold, which included velocities within the variability of normal oscillations of balance (i.e. 0.0005 and 0.001 rad/s), the accompanying balance responses were not interpreted as imposed ankle perturbations. Overall, these results support the proposition that participants can use sensory cues (i.e. vestibular) that encode only the whole-body balance response in order to accurately perceive the direction of an external perturbation when focusing on their ankle movements.

## Discussion

In the present experiments, we established the mechanisms underlying our conscious sense of standing balance by quantifying perceptual thresholds of whole-body and ankle perturbations inducing context-dependent ambiguous and unambiguous cues of self-motion. When standing immobile, participants perceived whole-body (0.0015 rad) and ankle perturbations (0.003 rad) only in the direction of the imposed motion, with a perceptual threshold of 0.0016 ± 0.0004 rad/s and 0.0023 ± 0.0012 rad/s, respectively. When balancing upright, whole-body perturbations with velocities ≥ 0.0005 rad/s always elicited a corrective balance response in the direction opposite to the imposed motion. Consequently, participants experienced two consecutive motions in opposite directions: first the applied perturbation followed by their own balance response. For whole-body perturbation velocities (0.00025 to 0.0025 rad/s) below or near the variability in natural whole-body oscillations (∼0.0022 rad/s), participants were not aware that their balance system detected and responded to the imposed whole-body motion, and they perceived their own balance response as imposed motion. This resulted in their perception performance being better explained by two processes of perception: one for detecting the consequences of their balance response at small velocities (threshold: 0.00045 ± 0.0003 rad/s) and one for detecting the imposed perturbation at larger velocities (threshold: 0.0036 ± 0.0003 rad/s). Finally, although ankle perturbations also evoked corrective responses when balancing upright, participants instructed to focus on their ankle movements unambiguously perceived the ankle perturbation only in the direction of the imposed motion at a perceptual threshold of 0.0073 ± 0.0034 rad/s. Overall, our results reveal how actions of the balance motor system can mislead the conscious sense of balance. Standing humans may perceive their own balance response as imposed motion when perturbation velocities are within the natural variability of balance (i.e. 1 SD) but only when self-motion cues arising from balance responses and imposed motion are encoded ambiguously.

### Misattribution of the causality of self-motion is driven by unperceived actions of the balance system

When participants were exposed to whole-body perturbations while balancing (Experiment 1), we observed a balance response in the direction opposite to the imposed perturbation for all trials with perturbation velocities ≥ 0.0005 rad/s and an approximate threshold for eliciting a balance response of ∼0.00025 rad/s (i.e. whole-body motion opposite to the perturbation identified in 70 ± 8% of trials). This threshold for evoking balance responses was ∼84% smaller than the perceptual threshold when participants were immobile (0.0016 ± 0.0004 rad/s), suggesting that the balance system is better at detecting (and responding to) whole-body movements than the participant’s conscious awareness of them. Despite generating their own balance responses, participants erroneously perceived some of these self-generated motions as imposed perturbations. Indeed, participants mostly reported the direction of their balance response (i.e. opposite direction to the imposed perturbation) for whole-body perturbation velocities ranging from 0.0005 to 0.0025 rad/s (Fig. 2B). These results suggest that humans standing upright freely may have minimal or absent perception of very small imposed motion below the natural variability of balance *and* the elaboration of a corrective balance response to this perturbation (8, 13, 17). These results overturn the longstanding view that a single representation of the body and world is relied upon for both the sense and control of balance (4–7). Instead, the balance system can detect and respond to external disturbances outside of perceptual awareness, and these actions can lead to perceptual misattributions of the causality of movements.

The lack of awareness of the motor actions of our balance control system (despite the perception of their sensory consequences) may also explain why standing balance feels mostly effortless until a pathology impedes its control. In support of this view, indirect physiological evidence corroborates the minimal or absent perception of the motor actions of our balance control system: motor commands producing most of the torque required to maintain upright balance induce minimal blood pressure responses and limited cortical activity compared to similar voluntary motor commands (9). Furthermore, our conscious perception of both self-balancing and head orientation functions separately from the motor actions of balance control (10, 11). These observations provide converging evidence that our balance system corrects our posture using its own sensorimotor principles without our awareness because we do not know we generated these actions and only perceive their resulting sensory consequences. As such, the dissonance between perceptual awareness and ongoing self-balancing actions are reminiscent of the differential processing of visual information for action or perception (31, 32). For instance, participants asked to lift large and small objects of equal weight misjudge the smaller to be heavier (i.e. the size–weight illusion), even though the motor actions are appropriately scaled to the true object weight (33). The findings in the current study extend beyond a simple dissociation of action and perception for balance, showing that not only do self-generated balance actions operate independent from awareness, but they can interfere with perception of self-motion to the point of attributing our own actions to an external event.

**Fig. 4.**
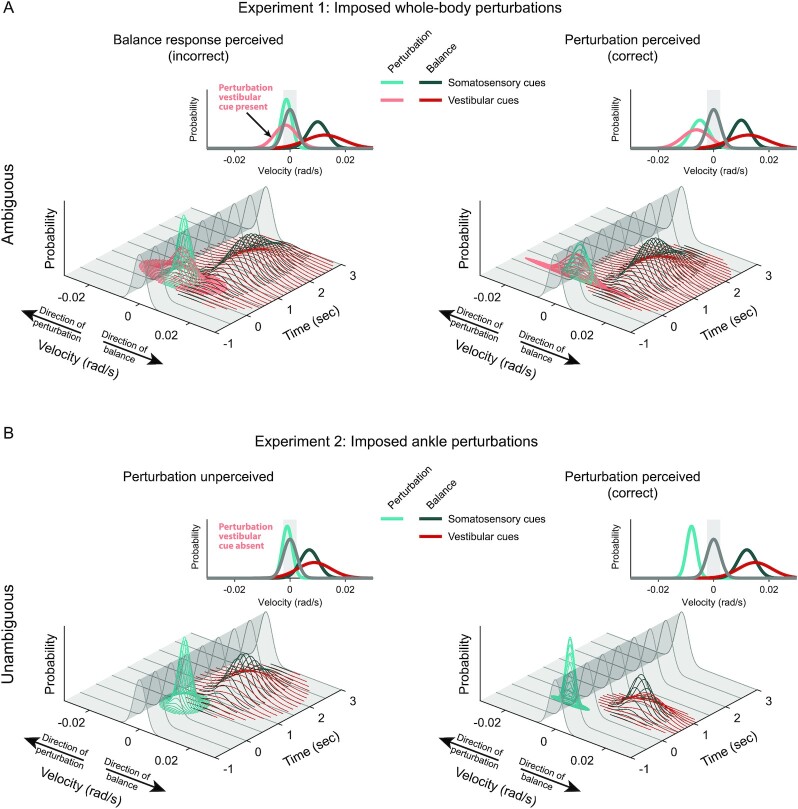
Conceptual model of sensorimotor integration for the perception of balance. The model explains how ambiguous and unambiguous encoding of imposed and balance-correcting self-motion, relative to the natural variability of standing, cause participants to perceive the imposed motion either in the direction of the balance response or the perturbation. (A) The ambiguous signaling of whole-body perturbations and accompanying balance responses caused participants instructed to focus on their whole-body motion to (incorrectly) perceive the imposed motion in the direction of the balance response (left—*balance response perceived*). This occurred only when the sensory cues in the direction of the perturbation were within the natural variability (i.e. 1 to 2 SD) of sway velocity (gray distribution) but not when they exceeded the natural variability (right—*perturbation perceived*). The plotted distributions include the natural variability of standing (gray), the vestibular (light red) and somatosensory (light blue) cues elicited by the imposed perturbation, and the vestibular (dark red) and somatosensory (dark blue) cues elicited by the balance response. Distributions for somatosensory cues are narrower than vestibular cues based on the lower thresholds reported during passive whole-body (i.e. vestibular) and ankle (i.e. somatosensory) motion (13). Insets show the projection of the maximum time-varying distributions and highlight conditions where cues of the perturbation fall within (“incorrect”) or outside (“correct”) 1 to 2 SD of natural sway variability (shaded gray region). (B) The unambiguous signaling of ankle perturbations caused participants instructed to focus on ankle motions to perceive motion only in the direction of the perturbation. Because vestibular cues of motion were only evoked by the balance response (i.e. vestibular cues elicited by the perturbation are absent), participants either perceived nothing (left plot) or the direction of the perturbation (right plot) when the accompanying sensory cues of the perturbation fell inside (“unperceived”) or outside (“correct”) the natural variability of standing (i.e. 1 to 2 SD, shaded gray region), respectively.

For larger whole-body perturbation velocities (i.e. 0.003 and 0.004 rad/s), participants mostly perceived motion in the direction of the perturbation. The threshold for perceiving these larger whole-body perturbations (0.0036 ± 0.0003 rad/s) was 1.6× larger than the natural statistics (i.e. 1 SD) of whole-body sway velocity (0.0022 ± 0.0005 rad/s). This suggests that participants may only be able to perceive the direction of imposed whole-body perturbations when their effect on the expected balancing behavior exceeds ∼90% of the statistical variability of normal standing (see conceptual model in Fig. 4A—perturbation perceived). At lower velocities, on the other hand, the perturbation’s influence on expected balance behavior remains within the variability of normal standing (Fig. 4A—balance response perceived), and as a result, participants are unable to detect that motion. These results align with the prediction that variability in whole-body sway during quiet standing is in part due to errors in our estimates of self-motion (34). Given that our brain relies on the accuracy of these estimates to identify unexpected movements (1, 2, 35, 36), there is a low probability that the imposed motion can be perceptually extracted from self-generated balancing motion when a perturbation applied to the whole-body remains within the natural variability (or error) of our own motion. This is also likely the reason that the threshold for perceiving the direction of the imposed whole-body and ankle perturbations during Balancing conditions was about two to three times larger than the threshold identified in the Immobile condition. Indeed, even at the highest perturbation velocity in both Experiments, participants had a lower rate of perceiving the direction of the imposed perturbation in the Balancing compared to the Immobile condition (82% versus 95% at 0.004 rad/s in Experiment 1 and 90% versus 99% at 0.02 rad/s in Experiment 2; Figs. 2 and 3). This finding may hold clinical relevance for aging and populations with certain pathologies (e.g. vestibular and cerebellar patients), where the expected increase in sensory and motor noise could widen the natural variability of standing and cause misattributions of self-motion at larger perturbations that may threaten stability. Furthermore, our findings may relate to how humans interact with mechatronic mobility systems (e.g. Segway’s, electric unicycles, or exoskeletons); the learned predictions of the device’s closed-loop interaction with the balance system may lead to ambiguities in the perception of self-motion. Future experiments that carefully manipulate both the natural variability of standing balance and the dynamics of the support surface may reveal how noise or ambiguity added to the brain’s estimate of whole-body motion influences perceptual thresholds and the potential for falls.

Finally, our observations of self-motion perception during balance are also relevant to the sense of agency; i.e. the conscious sense of being the author of one’s own action (3, 37, 38). Agency of our own actions is thought to occur when the predicted sensory consequences of our motor commands (generated through an internal representation of body dynamics) matches the actual sensory feedback (37, 39–41). However, because in our experiments participants were tasked to report the direction of the perturbation, as opposed to their own balancing action, our results may better represent the phenomena of nonagency (i.e. “this is not my action”) (3, 42, 43), especially when the corrective responses produced by the balance system arise without conscious awareness. Indeed, these misattributions of self-generated motion may be more akin to patients with schizophrenia who can have difficulty attributing their own actions to themselves (44–46) and demonstrate in healthy individuals how motor actions can lead to abnormalities in our awareness of movement causation.

### Ambiguous cues of whole-body motion cause participants to erroneously perceive their balance response as imposed motion

When participants (incorrectly) perceived the whole-body perturbation in the direction of their balance response, angular displacement and velocity were larger in the direction of their balance responses compared to when they perceived the direction of the imposed perturbation (Fig. S2B). As a result, it may be possible that participants perceived their own self-generated motions as an imposed perturbation through mechanisms similar to backward masking [see review (47)]. This phenomenon, commonly used to explore the dynamics of visual information processing, occurs when a more intense visual stimulus—immediately following a small stimulus—blocks the conscious perception of the small stimulus. When participants were exposed to whole-body perturbations, the balance response was 3 to 17× larger than the perturbation velocity and 4 to 12× larger than the variability (SD) of natural sway, regardless of the participants’ perception. During ankle perturbations, however, we saw no evidence of backward masking from the balance response onto the participant’s perception of the direction of the imposed motion. Instead, participants only reported the direction of the imposed ankle motion, despite the velocity of their balance response being 1 to 12× larger than the perturbation velocity and 4 to 14× larger than the variability (SD) in natural sway. Ankle perturbations target specifically ankle and feet somatosensory cues and, consequently, the resulting whole-body balance correcting responses can be disambiguated from the ankle perturbation because vestibular cues encode only the balancing movement (Fig. 4B). This is in stark contrast with whole-body perturbations where the brain ambiguously encodes self-motion of the imposed disturbance and the subsequent balancing action using the same sensory cues. Therefore, a more likely explanation is that because participants are mostly unaware of having generated their own balance corrections, the requirement to perceive their whole-body motion coupled with the ambiguous encoding of self-motion cues led participants to interpret their corrective responses as imposed motion (Fig. 4A—balance response perceived).

To further explain the perception of the sensory consequences of balance as imposed whole-body perturbations, we proposed a model estimating the weights assigned to a process detecting ambiguous cues encoding the direction of the imposed perturbation and the subsequent direction of the balance response (Fig 2C). The model’s relative weights estimate the likelihood of favoring one motion over the other one for the perception of imposed whole-body motion. Our model revealed that perception of the direction of the balance response is more likely when the perturbation velocity is smaller than 0.00275 rad/s whereas perception of the perturbation direction is more likely for perturbation velocity larger than 0.00275 rad/s. This transition aligned with natural variability of standing balance identified in the 1.5 s preceding the perturbation (SD: 0.0022 ± 0.0006 rad/s). Consequently, for all whole-body perturbation velocities, freely standing humans have to choose from shared sensory cues that indicate two consecutive and oppositely directed whole-body motions and favor one or the other depending on the velocity of the imposed perturbation relative to the natural statistics of balance. Indeed, even for the largest perturbation velocity used in Experiment 1 (0.0040 rad/s), the weights for the function representing detection of the imposed perturbation did not reach 1, suggesting participants combined sensory information from opposing motion directions to decide which motion was imposed.

### Perception thresholds

In the Immobile condition, perceptual performance to whole-body perturbations (Experiment 1) was appropriately represented by a single psychometric curve, with a threshold for perceiving the direction of the perturbation of 0.0016 (± 0.0004) rad/s. This threshold is similar to a threshold identified when the whole-body was immobilized and participants balanced an external inverted pendulum with sensory cues limited to somatosensory feedback [0.0018 rad/s (13)], but smaller than thresholds identified when the whole-body was passively moved from an upright posture with only vestibular feedback available: ∼0.01 rad at 0.004 rad/s (13) and 0.087 rad at 0.0008 rad/s (18). Because our participants could use somatosensory and vestibular cues to detect the perturbation direction, the threshold identified in the Immobile condition corresponds to the one reported by Fitzpatrick and McCloskey (13). In the Balancing condition, participants primarily reported the direction opposite to the imposed whole-body perturbation for small velocities (≤0.0025 rad/s) and the direction of the imposed perturbation for larger velocities (>0.0025 rad/s). The threshold for detecting the direction of the balance response to the imposed perturbation (i.e. incorrect detection of the perturbation direction) in the Balancing condition was 0.00045 (±0.0003) rad/s, which was further confirmed by our model’s estimated weights for detecting the balance response (0.00057 rad/s). This identified threshold is small compared to other thresholds previously reported in the literature (13, 16). It is also approximately three and eight times smaller than the thresholds for detecting the direction of the perturbation identified for the Immobile (0.0016 ± 0.0004 rad/s) and Balancing (0.0036 ± 0.0003 rad/s) conditions, respectively. As discussed above, participants experienced larger whole-body motion in the direction of their balance responses than in the direction of the imposed perturbations and because they were not aware that their balance system generated these actions, they were more likely to detect them on top of their postural oscillations.

For the Immobile and Balancing conditions during ankle perturbations (Experiment 2), the rate of correct perception was best represented by a single psychometric function, reaching thresholds for perceiving the direction of the perturbation of 0.0023 ± 0.0012 rad/s and 0.0073 ± 0.0034 rad/s for Immobile and Balancing conditions, respectively. The threshold identified during the Immobile condition corresponds to estimates from previous studies under equivalent conditions (13, 14). Similarly, the threshold identified during the Balancing condition, though approximately three times larger than in the Immobile conditions, was comparable to those reported by Thelen et al. (17), where perturbations were applied to a single foot while participants balanced freely (0.0073 rad/s at 0.003 rad versus 0.0087 rad/s for 0.0022 rad). By combining both conditions in a single experiment, we reconcile these large discrepancies in the literature, and reveal that previous estimates of somatosensory thresholds during immobile standing substantially overestimate the ability of participants to perceive imposed ankle motion during standing balance. As noted above, a likely explanation for these differences may be the uncertainty associated with detecting small ankle perturbations when they fall within the natural variability of ankle movement during normal balancing oscillations.

Our main results of perceiving the sensory consequence of balance as imposed perturbations have important practical and theoretical consequences for the estimation of individual sensory thresholds involved in the perception of whole-body upright motion (13). The multisensory fusion processes underlying the control of balance may be difficult to characterize due to the ambiguity in coding imposed perturbations and balance responses of whole-body self-motion. Although this difficulty may not be apparent for perturbations targeting certain sensory cues (see ankle perturbation results for Experiment 2), it may confound the interpretation of perception to imposed motions targeting multisensory integration (whole-body perturbations used in Experiment 1). A critical outcome of the present experiments relates to the identification of thresholds to evoke a self-correcting balance response under varying conditions. These balance-related motion thresholds appear to be largely under the control of nonperceptual processes governing upright stance and always in response to the perturbation stimuli. Hence, carefully designed mechanical or sensory stimuli will enable the characterization of bias and variability in individual cues needed to model and predict the sensorimotor integration processes regulating standing balance.

### Limitations

Although we controlled the participants’ position and velocity in the Balancing condition before perturbation onset (see the “Materials and methods” section), we could not immobilize their posture (i.e. zero velocity) to trigger the perturbation at their exact preferred angle. Therefore, the initial angle and velocity at which the perturbation was triggered varied, which may contribute to perception performance variability in the Balancing condition for both whole-body and ankle perturbations. This limitation, however, was necessary to assess the perceptual processes underlying upright stance in a freely balancing task. Furthermore, restricting the natural variability of balance, through imposed or volitional means, may lead to underestimated perceptual thresholds of our normal standing behavior.

## Conclusions

When exposed to whole-body or ankle perturbations while balancing upright, human participants generate compensatory balance responses driving their whole-body in the direction opposite or identical to the perturbation, respectively. During whole-body perturbations, participants remain largely unaware they generated these motor actions, but perceive their sensory consequences and interpret self-generated motion as an imposed motion when the magnitude of the applied perturbation remains below the natural variability of whole-body sway. Because the available sensory cues of balance ambiguously encode both the direction of the perturbation and the balance response, the successive self-motion of the whole-body in both directions can cause misattributions of self-generated actions as external events. In contrast, participants exposed to ankle perturbations use sensory cues that encode only the balance-correcting response to unambiguously distinguish the direction of ankle rotations evoked by a perturbation. These results show that our sense of balance involves two mechanisms that may interfere: a first threshold for the balance control system to detect, integrate, and respond to a whole-body perturbation, and a second (larger) threshold for perceiving the direction of an imposed perturbation. Overall, our study establishes that humans can misattribute their own corrective balance response as imposed motion when the sensory cues of self-motion from balance responses and imposed perturbations are ambiguous and perturbation velocities are within the natural variability of standing balance.

## Materials and Methods

### Participants

Twenty healthy adults without any history of neurological or muscular disorders participated in two separate experiments (13 males, mean age: 26.4, SD: 2.6 y old) for this study. The experimental protocols were approved by the University of British Columbia Clinical Research Ethics committee (Experiment 1) or the Medical Ethics Committee of the Erasmus MC Rotterdam (Experiment 2) and conformed to the *Declaration of Helsinki*, except for registration in a database. Each participant completed only one experiment (i.e. *N* = 10 in Experiment 1 and *N* = 10 in Experiment 2) and experimental protocols were explained to the participants prior to obtaining their written informed consent.

### Experimental setup

Two experiments were conducted to study the mechanisms underlying our conscious sense of standing balance by assessing perception thresholds to imposed whole-body and ankle perturbations. Participants stood upright on robotic apparatuses designed to simulate the control of standing balance with the mechanics of an inverted pendulum restricted to anterior–posterior motion (Fig. 1) (20, 27–29). The robots applied the self-generated (i.e. balance simulation driven motion corresponding to the participants’ applied ankle torque) and imposed rotatory motion of the whole-body (Experiment 1) about the ankles via the backboard actuated by a motor. Imposed rotary motion of the support surface (Experiment 2) was applied about the ankles via the footplate actuated by a second motor.

### Protocol

In Experiment 1, we applied whole-body perturbations while participants stood immobile or balanced freely and asked them to report the direction of the imposed motion (forward and/or backward). In Experiment 2, we applied ankle perturbations while participants stood immobile or balanced freely and asked them to report the direction of the imposed support-surface motion (toes-up and/or toes-down). For each experiment, two sessions were performed on different days to assess upright perceptual thresholds while immobile and balancing. Participants were told that perturbations would be delivered through motion of either the whole-body (Experiment 1) or the support-surface (Experiment 2). Participants were instructed to report the direction of any imposed movements and in the case they did not perceive any imposed movement, they were required to make their best guess (forced-choice protocol).

During the Immobile condition, participants were kept in their preferred upright posture while strapped to the backboard of the robot when the perturbations were delivered. During the Balancing condition, participants were instructed to hold a steady position close to their preferred posture while they maintained upright standing balance on the robot. A perturbation was initiated only when participants were within 1 SD of their previously measured preferred posture for a period of approximately 1.5 s and the backboard velocity was near 0. More details are given in the Supplementary Material Appendix.

### Data analysis

For each trial, the direction of self-motion reported by the participants was recorded and identified as “correct” or “incorrect,” relative to the direction of the imposed perturbation. To estimate the perception performance of each participant, the proportion of correct answers was calculated individually for each perturbation velocity in each condition (Immobile and Balancing) from both experiments. A perception performance above 70% correct would indicate that participants reported the direction of the perturbation, whereas a perception performance below 30% correct would indicate that participants reported the direction opposite to the imposed perturbation. To describe each participant’s perception performance of whole-body perturbations in both Immobile and Balancing conditions, we estimated the function best fitting their results using psychometric curves. Two psychometric functions were used to explain the data related to the perception of imposed self-motion: a single psychometric curve (based on a Gaussian cumulative distribution function) and a dual psychometric curve (based on the combination of a normal and inverted Gaussian cumulative distribution function). In each condition, and for each participant, data were fit with both the single and the dual psychometric curves using a Bayesian estimation approach. To assess the fitting accuracy of each psychometric curve to capture the experimental results, we computed the AIC and BIC.

Based on the results from the AIC and BIC, we used the best-fitting psychometric function found in each condition to estimate each participant’s perception thresholds. For each participant, the threshold for perception of the direction of the perturbation was defined as the smallest velocity where the function reached 70% of correct perception (13, 14), in both conditions. To identify a threshold for perception of the direction opposite to the perturbation (Experiment 1), we extracted the smallest velocity where the function reached 30% of correct perception (i.e. 70% of perception in the direction opposite to the direction of the imposed perturbation). In the Balancing condition of Experiment 1 (see the “Results” section), the dual psychometric curve fitting was used to characterize the structure of the data and identify thresholds but did not capture the requirement for participants to weigh sensory signals successively encoding their self-motion to the imposed movement and their own balance response to the perturbation. To improve the physiological relevance of our curve fitting in the Balancing condition, we computed the distribution of the perceptual performance as a weighted model combining probabilities of perceiving motion in the direction of either the imposed perturbation or the balance response. This model was constructed on the averaged perception performance of all participants using two probability distributions and two weighting functions (see the Supplementary Material Appendix).

In the Balancing condition, we also quantified the proportion of trials where participants generated a whole-body motion in the opposite direction to the imposed perturbation for each perturbation velocity (see Supplementary Material Appendix for more details). We quantified the peaks of whole-body angular position and velocity to examine how the whole-body motion experienced by participants compared to the statistics of the balance oscillations over the same period leading up to perturbation onset (i.e. SDs of position and velocity 1.5 s before perturbation). These data were also used to determine whether this whole-body motion was different between trials where they (*correctly*) perceived the direction of the imposed perturbation and trials where they (*incorrectly*) perceived the opposite direction to the imposed perturbation. More details are given in the Supplementary Material Appendix. See also ref. (48) for available data.

### Statistics

All normally distributed data in the “Results” section and figures are expressed as means ± 1 SD and non-normally distributed data are expressed as medians/interquartile ranges, unless otherwise specified. Statistical analyses were performed with RStudio or Matlab and an *α* level of 0.05 was set for significance. All details regarding statistical tests performed are given in the Supplementary Material Appendix.

## Supplementary Material

pgac174_Supplemental_File

## Data Availability

Data and code needed to generate the result figures can be found on Borealis at https://doi.org/10.5683/SP3/BSZCOO.
