# Correction to: Unperceived motor actions of the balance system interfere with the causal attribution of self-motion

**DOI:** 10.1093/pnasnexus/pgad039

**Published:** 2023-02-15

This is a correction to: Romain Tisserand, Brandon G Rasman, Nina Omerovic, Ryan M Peters, Patrick A Forbes, Jean-Sébastien Blouin, Unperceived motor actions of the balance system interfere with the causal attribution of self-motion, *PNAS Nexus*, Volume 1, Issue 4, September 2022, pgac174, https://doi.org/10.1093/pnasnexus/pgac174

In the originally published version of this manuscript, one of Patrick A Forbes's affiliations was inadvertently omitted. The omitted affiliation is Department of Biomechanical Engineering, Delft University of Technology, Delft, The Netherlands.

This error has now been corrected.

