# Supplementary material for: Unperceived motor actions of the balance system interfere with the causal attribution of self-motion
Source: PNAS Nexus. 2022 Aug 27;1(4):pgac174. doi: 10.1093/pnasnexus/pgac174 (PMC9802180; doi:10.1093/pnasnexus/pgac174)
Supplement: pgac174_Supplemental_File [file pgac174_supplemental_file.docx]

**
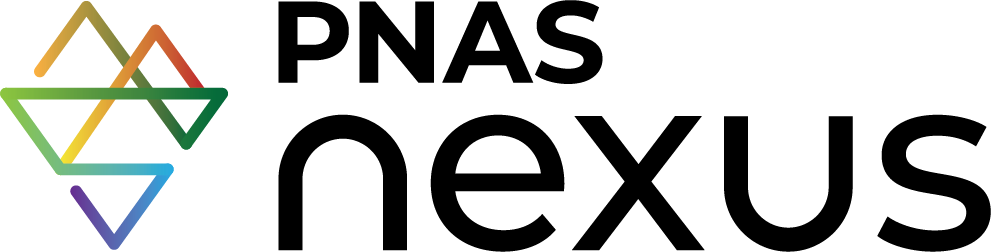
**

**Supplementary Information for**

Unperceived motor actions of the balance system interfere with the causal attribution of self-motion

Romain Tisserand, Brandon G. Rasman, Nina Omerovic, Ryan M. Peters, Patrick A. Forbes*, Jean-Sébastien Blouin*

*Senior authors contributed equally

Patrick A. Forbes

Email: [p.forbes@erasmusmc.nl](mailto:p.forbes@erasmusmc.nl)

Jean-Sébastien Blouin

Email: [jean-sebastien.blouin@ubc.ca](mailto:jean-sebastien.blouin@ubc.ca)

**This PDF file includes:**

Supplementary text

Figures S1 to S3

Tables S1 to S2

SI References

Supplementary Information Text

SI Materials and Methods

**Experimental set-up.** Two experiments were conducted to study the mechanisms underlying our conscious sense of standing balance by assessing perception thresholds to imposed whole-body and ankle perturbations. In Experiment 1, we examined how imposed whole-body perturbations and motor actions of the balance system interact to induce ambiguous cues of self-motion that shape the conscious perception of balance. We applied whole-body perturbations while participants stood immobile or balanced freely and asked them to report the direction of the imposed disturbance. In Experiment 2, we applied ankle perturbations while participants stood immobile or balanced freely to examine whether the perception of the imposed ankle motion can be disambiguated from the balance response when certain sensory cues (i.e., vestibular) only encode the balance-correcting response.

Participants stood upright on robotic apparatuses designed to simulate the control of standing balance with the mechanics of an inverted pendulum restricted to anterior-posterior motion (Fig 1) (1-4). The mechanical load of the upright body was simulated using a real-time motion controller (PXI-8108 [Experiment 1] or PXI-8880 [Experiment 2] Motion Controller, National Instruments, TX, USA), running at 2000 Hz (Experiment 1) or 500 Hz (Experiment 2). We inputted the mass and height of the center of mass for each participant as parameters of the robotic balance simulation. The mass of the participants was computed using the vertical component of the reaction force on a force plate (AMTI OR6-7 [Experiment 1] or AMTI BP400x600 [Experiment 2] Watertown, MA, USA). To measure the height of their center of mass, participants laid supine on a rigid board balanced over a round tube positioned transversally under the board and shifted their body longitudinally over the board until the distribution of their mass balanced it. The distance between their lateral malleolus and the tipping point was determined as the height of the participant’s center of mass (average 0.92 ± 0.07 m). Participants controlled the real-time balance simulations by applying ankle plantar/dorsiflexor torque on the force plate, while being braced to a backboard. The robots applied the self-generated (i.e., balance simulation driven motion corresponding to the applied ankle torque) and imposed rotatory motion of the whole-body about the ankles via the backboard actuated by a motor (SGMCS-2ZN3A-YA21, Yaskawa, Japan; maximum continuous torque: 600 Nm; angular resolution of 0.00034° [Experiment 1] or ECMA-J11020S4, Delta, Taiwan; maximum continuous torque: 6170 Nm; angular resolution of 0.0000054° [Experiment 2]). Imposed rotary motion of the support surface (Experiment 2) was applied about the ankles via the footplate actuated by a second motor (ECMA-C10604SS, Delta, Taiwan; maximum continuous torque: 234 Nm; angular resolution of 0.000014°). To maintain a forward leaning position (-θ) on the robot, participants produced a plantarflexion torque (+T). Any increase in plantarflexion torque from a quasi-static posture induced a backward angular acceleration of the whole-body and reversely for a net dorsiflexor ankle torque, in the same way ankle torque stabilizes the body during normal over-ground standing (1,5). The range of the robot-induced balance motion was limited to 6° (0.10 rad) forward and 3° (0.05 rad) backward using software limits. Additional physical limits were positioned at ± 10.5° from vertical to ensure participants’ safety. All participants were able to maintain standing balance without difficulty and none of them ever reached software limits. All force plate data were amplified (× 4000) and acquired at 2000 Hz (Experiment 1) or 500 Hz (Experiment 2) (PXI-6289, National Instruments, TX, USA).

**Protocol.** Participants stood barefoot on the force plate and aligned their ankles with the backboard axis of rotation. Their feet position and orientation were marked with adhesive tape to ensure a reproducible feet position between trials and conditions. Participants were strapped to the backboard using two seatbelts (Fig 1) and foam covered their upper body to minimize any vibration from the backboard and to distribute the pressure from the seatbelts. Participants were blindfolded and wore earplugs (NRR32 – 32 decibels disposable earplugs, Stanley, CA, USA) and noise cancelling headphones (QC25 Acoustic Noise Cancelling Headphones, Bose, MA, USA [Experiment 1]; WH-1000XM3 Noise Cancelling Headphones, Sony, Japan [Experiment 2]) to minimize visual and auditory cues associated with movements of the robot, respectively, thereby isolating whole-body motion cues to vestibular and somatosensory feedback. Room lights were also turned off during the experiment.

*Experiment 1.* In Experiment 1 we assessed whether and how imposed whole-body perturbations and balance generated self-motion interact to induce ambiguity in the conscious perception of standing balance. Whole-body perturbations were applied at nine different velocities (0.0001, 0.00025, 0.0005, 0.001, 0.0015, 0.0020, 0.0025, 0.0030 and 0.0040 rad/s, respectively corresponding to 0.0057, 0.0143, 0.0286, 0.0573, 0.0859, 0.1146, 0.1432, 0.1719 and 0.2292 deg/s) at a fixed displacement (0.0015 rad; Fig 1). Given that somatosensory inputs are sensitive to both position and velocity (6,7), we chose to maintain a constant displacement while varying velocity. This approach is also commonly used to quantify perceptual motion thresholds during standing balance (8-12). Each perturbation velocity was applied 20 times, with 10 perturbations in the forward and 10 perturbations in the backward directions, for a total of 180 perturbations. The order of the perturbation velocity and direction was presented randomly. To ensure participants were ready and attentive to the imposed motion, the onset of a perturbation was indicated by an auditory tone (500 Hz, 250 ms) presented 300 to 500 ms before perturbation onset. Similarly, the end of a perturbation was indicated with a second tone (250 Hz, 250 ms), occurring 18 s after the perturbation onset, to ensure that the second tone would occur at least 3 s after the end of the slowest perturbation (*i.e.,* 15 s duration for the 0.0001 rad/s perturbation).

Two experimental sessions of 180 perturbations were performed on different days to assess upright perceptual thresholds while immobile and balancing (see Immobile and Balancing conditions described below). The order of these two experimental sessions was randomised between participants but they were aware of the condition they were experiencing. In both conditions, participants were told that only the whole-body would move. The specific instructions to the participant were: “to report any movement direction or directions (forward and/or backward) you feel was imposed between the two auditory tones. In the case you did not feel any imposed movement, we require you to make your best guess”. Participants were allowed to report the direction of as many imposed perturbations they perceived but they were forced to give their answer after the second tone (forced choice protocol). Despite experiencing only unidirectional perturbations, participants sometimes felt and reported two directions of imposed motion. On average, two directions of motion were reported in 6.8% of the Immobile trials and 15.3% of the Balancing trials. Participants reported a second direction mostly in the smallest (0.0001 rad/s) and the three largest velocities tested (from 0.0025 to 0.004 rad/s). No feedback regarding correct/incorrect responses was provided to the participants. To minimize fatigue, 5-minute breaks were given every 60 trials. Participants stepped off the robot and could either sit or walk around the experimental room. Before assessing the participants’ perception, we estimated their preferred posture during an initial trial where they swayed freely and asked to maintain standing balance on the robot for 2 minutes. From this initial trial, we computed their mean whole-body angle (mean and SD across participants: 0.0023 ± 0.0069 rad) and used this orientation as their preferred posture.

During the Immobile condition, participants were kept in their preferred upright posture while strapped to the backboard of the robot. The backboard, and therefore their whole-body, remained immobile until the onset of an imposed perturbation. Once the trial ended, participants were brought back to their upright preferred posture. Given that the participants’ ankle torque was not used to compute and update the whole-body angular displacement in the immobile condition, we further instructed participants to “let the backboard move them”.

During the Balancing condition, participants were instructed to hold a steady position close to their preferred posture while they maintained upright standing balance on the robot. We applied imposed perturbations to the backboard (*i.e.,* the participant’s whole-body) *on top* of the participants’ self-generated balance motion provided by the robot in response to their ankle torque production. Consequently, participants remained in control of their balance during the imposed whole-body perturbations: their ankle torque was used at all times to compute the backboard angle so that they did not feel any constrained movements during the constant velocity phase of the perturbation. Rather, the current protocol forced them to determine the direction of a whole-body perturbation inserted *inside* the balance control loop (Fig 1). Hence, balance-correcting responses to the whole-body perturbations were not prevented and no perceptible transition was present at perturbation onset/end. During the experiment, a perturbation was initiated only when participants were within one SD of their previously measured preferred posture for a period of approximately 1.5 sec and the backboard velocity was near zero. If participants were outside of these limits, they were verbally coached to their preferred posture before an imposed perturbation was initiated.

*Experiment 2.* In Experiment 2 we assessed whether ambiguities in the conscious perception of balance self-motion can be resolved when certain sensory cues encoding the balance response (e.g., vestibular) are not explicitly targeted by the imposed ankle rotation. Here, we delivered support surface perturbations that rotated the feet about the ankles at 7 different velocities (0.0005, 0.001, 0.002, 0.004, 0.008, 0.012 and 0.020 rad/s, which respectively correspond to 0.0286, 0.0573, 0.1146, 0.2292, 0.4582, 0.6875 and 1.1459 deg/s) at a fixed displacement of 0.003 rad. We doubled the displacement and increased the velocities of the ankle perturbations compared to the whole-body perturbations because pilot experiments indicated that even in immobile conditions, participants had difficulty detecting the smaller displacement used in Experiment 1. Also, the 0.003 rad imposed ankle displacement was similar to previous threshold estimates of ankle motion (11,13). Each perturbation velocity was applied 20 times in both the Immobile and Balance conditions, with 10 perturbations in the toes-up and 10 perturbations in the toes-down directions, for a total of 140 perturbations. Each condition was tested on separate days and the order of the perturbation velocity and direction was presented randomly. The same tones described in Experiment 1 were used to ensure participants were attentive to the perturbation, with the exception that the second tone was delivered 8 s after the perturbation onset because the slowest perturbation (0.0005 rad/s) had a duration of 6 s.

Procedures for the remainder of the experiment mimicked those outlined in Experiment 1 with some minor exceptions. First, in all sessions participants were told that only the support surface would move. The specific instructions to the participant were: “to report any movement direction or directions (toes-up/toes-down) you feel was imposed by the footplate between the two auditory tones. In the case you did not feel any imposed movement, we require you to make your best guess”. Although participants were allowed to report the direction of as many imposed ankle perturbations they perceived, all reported only one direction for all trials. Second, during the Immobile condition, participants remained strapped to the backboard (i.e., immobile) with their whole-body oriented at their preferred posture throughout the entire support surface perturbation. Participants were instructed to “let the support surface move their feet.” Third, during the Balance condition, participants remained in control of their whole-body balance at all times (and no whole-body perturbation was added to the control of balance) despite the change in support surface orientation that occurred throughout the ankle perturbation. A perturbation was initiated only when participants were within one SD of their preferred posture (mean and SD across participants: -0.0156 ± 0.0119 rad) for a period of approximately 1.5 sec and the backboard velocity was near zero.

**Data analysis**

For each trial, the direction of self-motion reported by the participants was recorded and identified as ‘correct’ or ‘incorrect’, relative to the direction of the imposed perturbation. If participants reported multiple consecutive directions during a trial, all directions were recorded but only the first reported direction was identified as correct or incorrect with respect to the perturbation direction because the imposed perturbation was the first experienced by participants. To estimate the perception performance of each participant, and therefore determine which direction they perceived, the proportion of correct answers was calculated individually for each perturbation velocity in each condition (Immobile and Balancing) from both experiments. A perception performance above 70% correct would indicate that participants reported the direction of the perturbation, whereas a perception performance below 30% correct would indicate that participants reported the direction opposite to the imposed perturbation.

*Experiment 1.* To describe each participant’s perception performance of whole-body perturbations in both Immobile and Balancing conditions, we estimated the function best fitting their results using psychometric curves. All fitting curves were parametrized as Gaussian cumulative distribution functions (CDF), each with a mean *µ* and a standard deviation *σ*. Because we hypothesized that participants would only perceive the direction of the imposed perturbation in the Immobile condition, we predicted their perception performance could be fitted with a single-psychometric function (i.e., 2 parameters). In the Balancing condition, however, we hypothesized that participants would perceive the opposite direction of the perturbation at small velocities and the direction of the perturbation at higher velocities. To explain the data related to a perception of two directions of self-motion, we predicted the perception performance could be fitted with a dual-psychometric function (i.e., 4 parameters). The dual-psychometric function was constructed with one function accounting for the probability of perception above chance level of 50% correct (direction of the perturbation) and another, inverted (‘1 – CDF’), function accounting for the probability of perception below chance level (direction opposite to the perturbation). These two curves were then summed together, point by-point, to obtain the final curve fit. In both conditions, all curves were constrained to the same range (values distributed from 0 to 0.004 rad/s).

In each condition, and for each participant, data were fit with both the single and the dual-psychometric curves using a Bayesian estimation approach. To assess the fitting accuracy of each psychometric curve to capture the experimental results, we computed the Akaike Information Criterion (AIC) and Bayesian Information Criterion (BIC), where in both measures a lower number indicates a better fit. These measures account for the different number of parameters used for each model (i.e., 2 for the single and 4 for the dual psychometric model). Multivariate curve fitting and model comparison described above were performed using LabVIEW 2018 (National Instruments, Austin, TX, USA) and Matlab (2018b version, Mathworks, Natick, MA, USA).

Based on the results from the AIC and BIC, we used the best-fitting psychometric model found in each condition to estimate each participant’s perception thresholds. For each participant, the threshold for perception of the direction of the perturbation was defined as the smallest velocity where the function estimated by the model reached 70% of correct perception (9,10), in both conditions. Because we hypothesized that participants would also perceive the motion of their balance response in the Balancing condition when cues were ambiguous, we identified a threshold for perception of the direction opposite to the direction of the perturbation for each participant. This threshold was defined as the smallest velocity where the function estimated by the model reached 30% of correct perception (*i.e.,* 70% of perception in the direction opposite to the direction of the imposed perturbation).

In the Balancing condition, the dual-psychometric curve fitting was useful to characterize the structure of the data and identify thresholds but did not capture the requirement for participants to weigh sensory signals successively encoding their self-motion to the imposed movement and their own balance response to the perturbation. To improve the physiological relevance of our curve fitting in the Balancing condition, we computed the distribution of the perceptual performance as a weighted model combining probabilities of perceiving motion in the direction of either the imposed perturbation or the balance response. This model was constructed on the averaged perception performance of all participants using two probability distributions and two weighting functions. The first distribution represents the probability of perceiving the direction of the imposed perturbation and was represented by a single-psychometric function ($'P\left( correct|Perturbation \right)'$). This distribution was obtained from the fit to the perception performance of all participants in the Immobile condition (see above). The second distribution represents the probability of perceiving the direction opposite to the direction of the perturbation and was represented by an inverted single-psychometric function (‘$P\left( correct|Balance response \right)'$). This distribution was optimized, together with the weighting functions (*ω_P_* and *ω_B_*), by fitting the weighted model (Eq. S1) to the perception performance of all participants in the Balancing condition (‘$P\left( correct|Balancing \right)'$).

| $P\left( correct \vert Balancing \right)=\omega_{P}\cdot P\left( correct \vert Perturbation \right)+\omega_{B}\cdot P\left( correct \vert Balance response \right)$ | (S1) |
| --- | --- |

Here, the corresponding weighting functions, $\omega_{P}$ and $\omega_{B}$ represent the probability that participants attribute the initial sensory cues to whole-body motion in the direction of the imposed perturbation or in the opposite direction of the imposed perturbation (i.e., the balance response), respectively. As a result, the weighting functions $\omega_{P}$ and $\omega_{B}$ sum to 1, with $\omega_{P}$ was modelled as a single sigmoidal CDF function and $\omega_{B}$ as a single, inverted, CDF (*i.e.,* ‘1 – CDF’) (Fig 2C). To estimate the six model parameters (the *μ* and *σ* for each of the $\omega_{P}$, $\omega_{B}$ and $P\left( correct | Balance response \right)$ functions)*,* we used a Bayesian estimation approach. We first modelled each participant’s perceptual behaviour in the balancing condition using a binomial equation (i.e., Bernoulli) and took the product across all 10 subjects to estimate the likelihood for each parameter combination. We then computed the posterior probability mass function across all parameter combinations using the likelihoods and a uniform prior according to Bayes rule to extract the best fit parameter values.

In the Balancing condition, we also quantified the proportion of trials where participants generated a whole-body motion in the opposite direction to the imposed perturbation for each perturbation velocity. We first identified the presence of a compensatory balance response when the first peak of the whole-body angular position in the direction opposite to the perturbation following perturbation onset was larger than the angular position averaged in the 1.5 s preceding perturbation onset ± 2 SD. In addition, we quantified the peaks of whole-body angular position and velocity to examine how the whole-body motion experienced by participants compared to the statistics of the balance oscillations over the same period leading up to perturbation onset (i.e., SDs of position and velocity 1.5 s before perturbation). These data were also used to determine whether this whole-body motion was different between trials where they (*correctly*) perceived the direction of the imposed perturbation and trials where they (*incorrectly*) perceived the opposite direction to the imposed perturbation. For each trial, we first removed the mean whole-body angle computed over the 1.5 s preceding perturbation onset. Then, angular velocity was computed as the first time derivative of the angular position, with a 2^nd^ order dual-pass digital Butterworth filter and a cut-off frequency of 20 Hz. On a trial-by-trial basis, we identified the first peak reached in the direction of the perturbation and the first peak reached in the direction opposite to the direction of the imposed perturbation (*i.e.,* direction of the balance response), both for the angular position and velocity. Each peak was then averaged across all trials from all participants and across perturbation velocities between 0.0005 and 0.0040 rad/s. Peaks measured for perturbation velocities of 0.0001 and 0.00025 rad/s were not included because perception performance was at a chance level and participants did not always produce a balance response (see Results). We expected the motion cues (i.e., angular displacement and velocity peaks) measured in the direction of the imposed perturbation to be larger when participants perceived the direction of the imposed perturbation (*i.e.,* correct response) compared to when they perceived the opposite direction of the perturbation (*i.e.,* incorrect response). In addition, we expected the angular displacement and velocity peaks measured in the direction opposite to the imposed perturbation (i.e., the balance response) to be smaller when participants perceived the direction of the imposed perturbation compared to when they perceived the opposite direction of the perturbation. Peaks were automatically identified using routines written in Matlab (2018b version, Mathworks, Natick, MA, USA).

*Experiment 2.* Data analyses for Experiment 2 followed similar procedures as those described for Experiment 1. Briefly, single and dual psychometric function models were fit to the resulting perception data. Because we hypothesized that participants would only perceive the direction of the imposed ankle perturbation in both the Immobile and Balancing conditions, we predicted their perception performance could be better fitted with a single-psychometric function. Data fit suitability for both conditions was quantified using the AIC and BIC. Using the best-fitting psychometric curve, we then extracted each participant’s perception thresholds of the imposed ankle motion (i.e., 70% correct responses). In the Balancing condition, we also examined the proportion of trials where participants produced a whole-body motion in response to the imposed ankle perturbation. This assessment served as a control to Experiment 1 to ensure that balance responses were present but that these could be separated from the imposed perturbation using sensory cues (i.e., vestibular) that only encode the balance-correcting response. Balance responses were again identified when the whole-body angular motion exceeded ± 2 SD above the average angular position in the 1.5 s preceding the perturbation. As in Experiment 1, these peak responses were also compared to the statistics of balancing oscillations over the same period leading up to perturbation onset (i.e., SDs of position and velocity in the 1.5 s before).

**Statistics.** To determine which psychometric curve was the most appropriate to fit the experimental data in each condition, we computed the AIC and BIC for both experiments. We compared these values across our two conditions for each experiment using a two-sample paired sign test because the data were not normally distributed (normality tested with a Shapiro-Wilk). For each participant, we identified thresholds from the psychometric functions for perceiving the direction of the perturbation (i.e., 70 % correct) both in the Immobile and Balancing conditions of Experiment 1 and 2, and a threshold for perceiving the opposite direction of the perturbation (i.e., 30% correct), but only in the Balancing condition of Experiment 1 (see Results). To determine whether the different thresholds (*i.e.,* perturbation velocities) identified from Immobile and Balancing conditions in Experiment 1 were different from each other, we compared them using a one-way repeated measures ANOVA, and *post-hoc* comparisons were performed using Tukey tests. A similar comparison of thresholds between Immobile and Balance conditions in Experiment 2 was performed using a Student’s t-test.

To link the participants’ perception performance with their whole-body motion during the Balancing condition in Experiment 1, averaged angular position and velocity peaks were compared between trials where participants perceived the direction of the perturbation (*i.e.,* correct) and trials where participants perceived the opposite direction of the perturbation (*i.e.,* incorrect). This comparison was performed using a two-way repeated measures ANOVA, with “Velocity” (seven levels: from 0.0005 to 0.0040 rad/s) and “Perception” (Correct and Incorrect) as factors. To decompose potential interactions and assess whether these peaks differed based on the applied perturbation velocity, we performed *post-hoc* Tukey tests.

Statistical analyses were performed with RStudio (version 1.0.153) or Matlab (version 2018b) and an α level of 0.05 was set for significance. All normally distributed data in the Results section and figures are expressed as means ± one SD and non-normally distributed data are expressed as medians/interquartile ranges (IQR).

SI Results


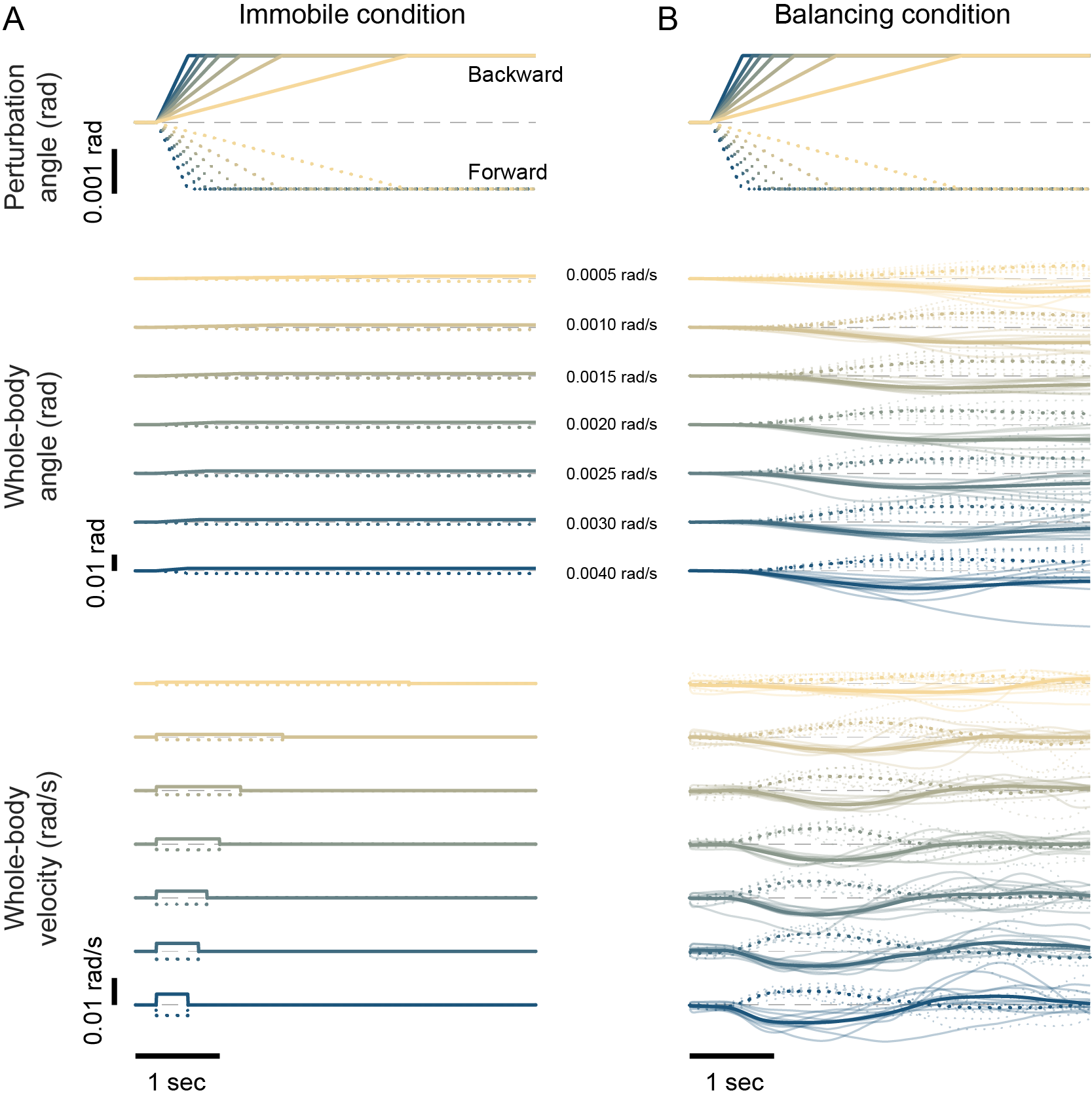


Fig. S1. Balance responses evoked by whole-body perturbations (Experiment 1). Imposed perturbation angle (top), whole-body angle (middle) and whole-body velocity (bottom) during the Immobile (A) and Balancing (B) conditions, respectively, for the seven largest perturbations. A positive angle corresponds to a backward motion whereas a negative angle corresponds to a forward motion such that solid lines represent backward perturbations and dotted lines represent forward perturbations. Note, during the Immobile condition in (A), whole-body angle and velocity followed the prescribed motion of the perturbation. During the Balancing condition in (B), larger whole-body angles and velocities were observed in the opposite direction to the perturbation. Thin lines represent the individual participants and thick lines represent the group average for each perturbation condition.


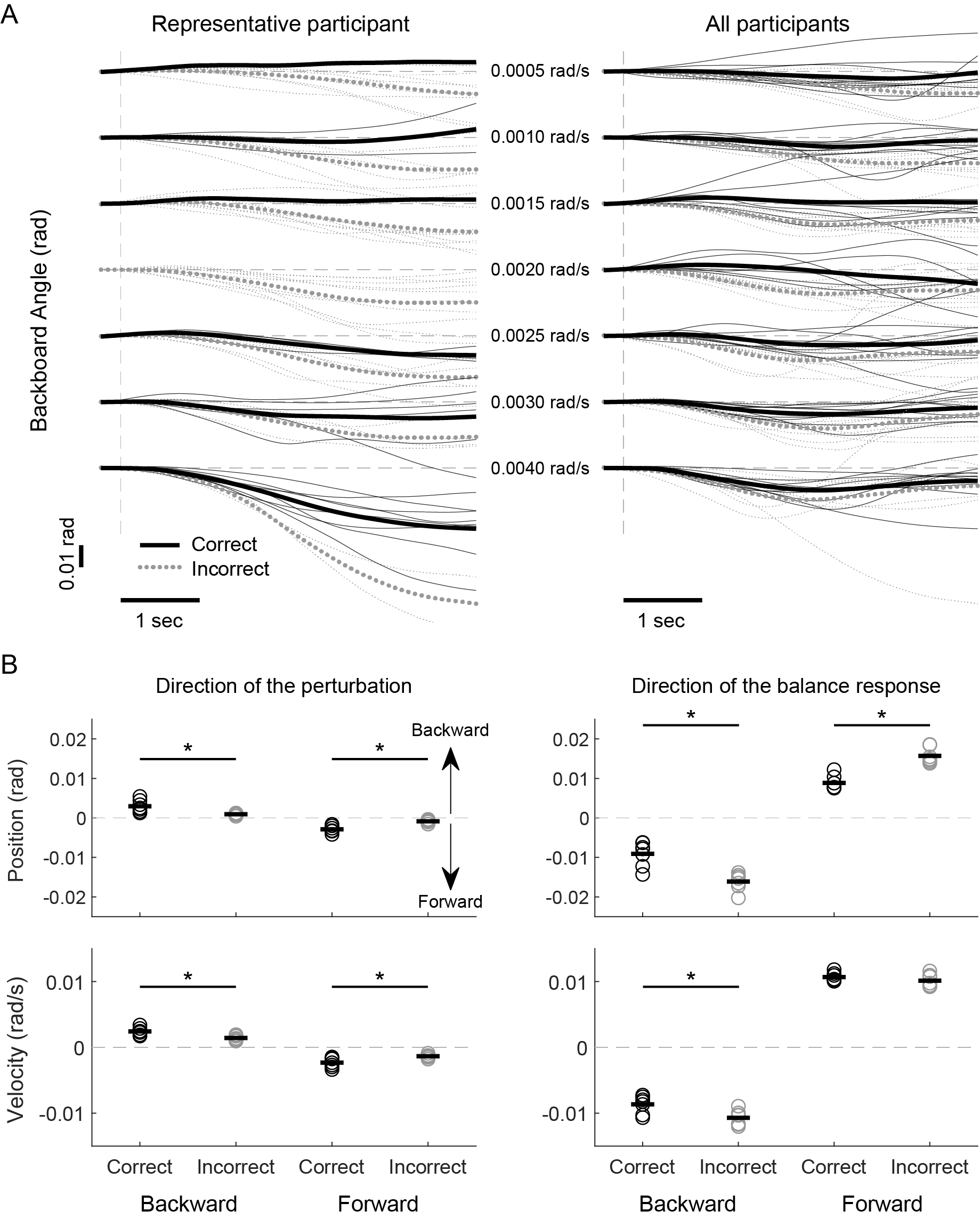


Fig. S2. Whole-body balance responses vary with perceptions of imposed perturbations during the balancing condition in Experiment 1. (A) Whole-body angle of a representative participant (left) and group means (right) during backward whole-body perturbations. Trials when participants 'correctly’ perceived the direction of the imposed perturbation are black traces and trials when they ‘incorrectly’ perceived the direction of the balance response are grey dotted traces. Thick lines in the representative participant plots (left) are the mean responses to the seven largest backward perturbations (i.e., from 0.0005 to 0.004 rad/s), while thin lines are individual trials where a balance response was evoked. Thick lines in the mean response plots (right) are the average displacements over all participants (n = 10) for those same perturbations, while thin lines are the average response of each participant. The peak whole-body angular displacement in the direction of the imposed perturbation and in the direction of the balance response were extracted from individual traces. Vertical dashed lines represent the onset of imposed motion. (B) Average values of the whole-body angular displacement and velocity peaks. Circles represent group-averaged (n = 10) responses at each of the seven largest velocity levels tested (i.e., from 0.0005 to 0.0040 rad/s) for correct (black circles) and incorrect (grey circles) trials. Each horizontal black bar represents the average whole-body angular displacement (top row) or velocity (bottom row) across the seven velocity levels where a balance response was observed. Data are presented relative to the perturbation direction (backward and forward) and the correctness of the participants’ perception (correct and incorrect). * indicates a p < 0.05. For all graphs, note that positive angle data indicates a movement in the backward direction and negative angle data indicate a movement in the forward direction. Angles have also been normalized so that 0 represents the participants’ initial preferred posture angle that participants maintained before the perturbation was delivered.


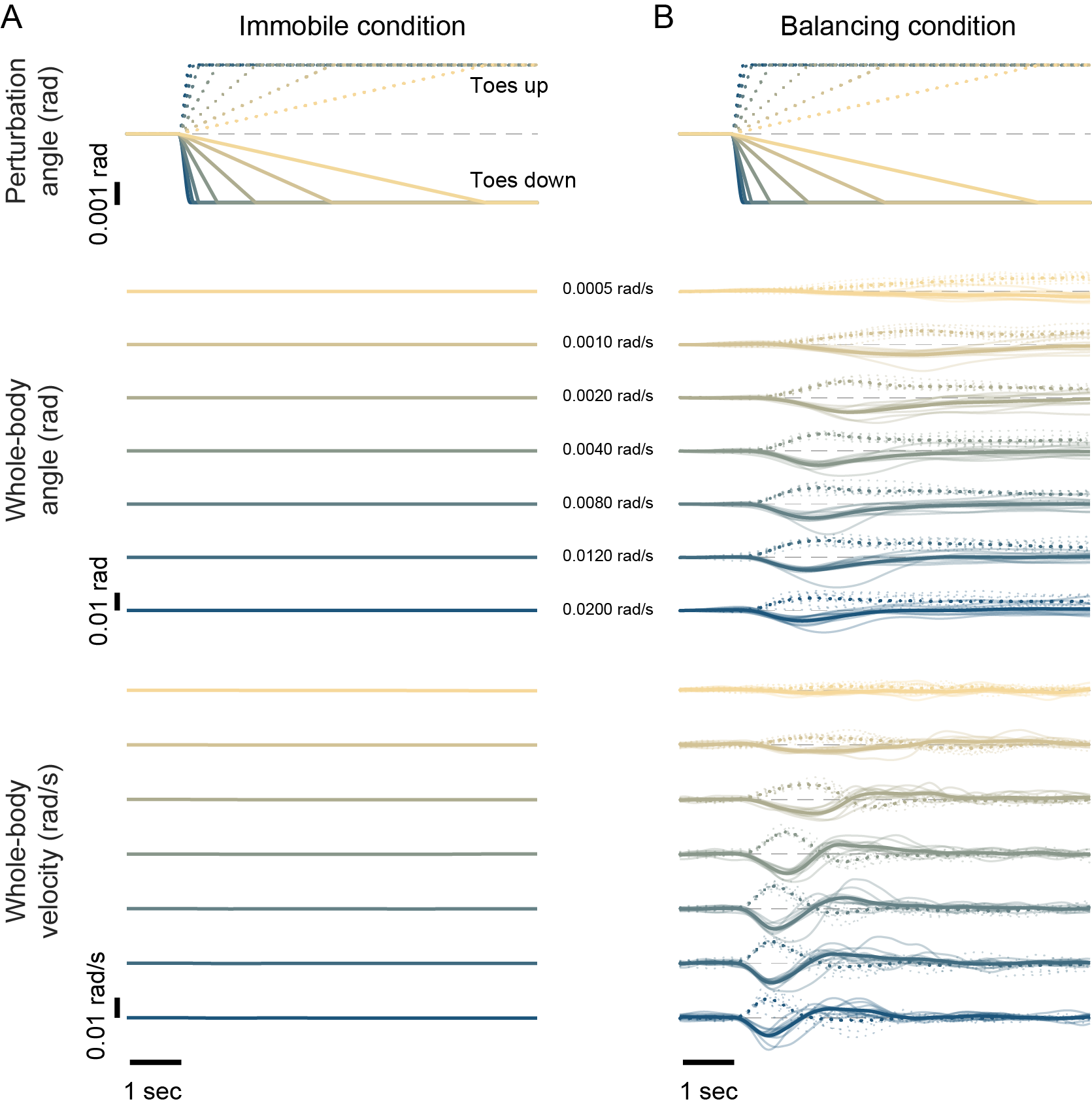


Fig. S3. Balance responses evoked by ankle perturbations (Experiment 2). Imposed perturbation angle (top), whole-body angle (middle) and whole-body velocity (bottom) during the Immobile (A) and Balancing (B) conditions, respectively, for all perturbations. A positive angle in the perturbation corresponds to toes up ankle movement (negative is toes down), while a positive angle in the whole-body motion corresponds to a backward motion (negative is forward). Solid lines represent toes down perturbations and dotted lines represent toes up perturbations. Note, during the Immobile condition in (A), whole-body angle and velocity were fixed throughout the perturbation. During the Balancing condition in (B), whole-body angles and velocities were observed in the same direction as the perturbation (i.e., toes down perturbations resulted in forward movements). Thin lines represent the individual participants while the thick solid lines represent the group average for each perturbation condition.

Table S1. Performance results of the psychometric curve fits to represent the perception performance in Immobile and Balancing conditions during Experiment 1 and 2. Statistical test outcomes (paired sign test) of the different comparisons are included. Data are expressed as medians/interquartile range.

| Variable | |  | Single-psychometric | Dual-psychometric | Z | p |
| --- | --- | --- | --- | --- | --- | --- |
| Experiment 1 | Immobile | AIC | 36.9/4.6 | 45.4/16.1 | 2.21 | 0.027 |
|  |  | BIC | 37.3/4.6 | 46.2/16.1 | 2.21 | 0.027 |
|  | Balancing | AIC | 68.5/7.3 | 44.9/10.7 | 2.21 | 0.027 |
|  |  | BIC | 68.9/7.3 | 45.7/10.7 | 2.21 | 0.027 |
| Experiment 2 | Immobile | AIC | 29.2/10.9 | 37.1/7.3 | 2.21 | 0.027 |
|  |  | BIC | 29.1/10.9 | 40.1/7.3 | 2.21 | 0.027 |
|  | Balancing | AIC | 32.3/5.8 | 39.0/7.9 | 2.21 | 0.027 |
|  |  | BIC | 32.2/5.8 | 38.9/7.9 | 2.21 | 0.027 |

Table S2. Whole-body kinematics (peak angular displacement and velocity) comparing trials where participants perceived the direction of the perturbation (i.e., correct) and trials where participants perceived the direction of the balance responses (i.e., incorrect) for Experiment 1. Statistical test outcomes (ANOVAs) of the different comparisons are also included. Forward and backward perturbation in the left column refer to the direction of the imposed perturbation. Values are reported as mean ± one SD.

|  | Correct | Incorrect | F | p |  |
| --- | --- | --- | --- | --- | --- |
| Peak angular displacement [rad] | | | | | |
| *In the direction of the perturbation* | | | | | |
| Forward perturbation | -0.0030 ± 0.0008 | -0.0008 ± 0.0003 | 52.77 | < 0.001 |  |
| Backward perturbation | 0.0030 ± 0.0009 | 0.0010 ± 0.0002 | 35.59 | < 0.001 |  |
| *In the direction of the balance response* | | | | | |
| Forward perturbation | 0.0089 ± 0.0018 | 0.0157 ± 0.0020 | 30.66 | < 0.001 |  |
| Backward perturbation | -0.0091 ± 0.0031 | -0.0161 ± 0.0022 | 31.06 | < 0.001 |  |
|  | | | | | |
| Peak angular velocity [rad/s] | | | | | |
| *In the direction of the perturbation* | | | | | |
| Forward perturbation | -0.0023 ± 0.0006 | -0.0013 ± 0.0003 | 23.38 | < 0.001 |  |
| Backward perturbation | 0.0024 ± 0.0006 | 0.0014 ± 0.0004 | 14.51 | < 0.001 |  |
| *In the direction of the balance response* | | | | | |
| Forward perturbation | 0.0107 ± 0.0006 | 0.0101 ± 0.0009 | 2.47 | > 0.05 |  |
| Backward perturbation | -0.0087 ± 0.0013 | -0.0107 ± 0.0011 | 8.62 | < 0.01 |  |

**SI References**

1. P. A. Forbes *et al.*, Transformation of vestibular signals for the control of standing in humans. *J. Neurosci.* **36**, 11510-11520 (2016).

2. T. P. Huryn, B. L. Luu, H. F. M. V. d. Loos, J. S. Blouin, E. A. Croft (2010) Investigating human balance using a robotic motion platform. in *2010 IEEE International Conference on Robotics and Automation*, pp 5090-5095.

3. B. L. Luu, T. P. Huryn, H. F. M. Van der Loos, E. A. Croft, J.-S. Blouin, Validation of a Robotic Balance System for Investigations in the Control of Human Standing Balance. *IEEE Trans. Neural Syst. Rehabil. Eng.* **19**, 382-390 (2011).

4. B. G. Rasman *et al.*, Learning to stand with unexpected sensorimotor delays. *eLife* **10** (2021).

5. B. L. Luu *et al.*, Human standing is modified by an unconscious integration of congruent sensory and motor signals. *J. Physiol.* **590**, 5783-5794 (2012).

6. U. Proske, S. C. Gandevia, The proprioceptive senses: their roles in signaling body shape, body position and movement, and muscle force. *Physiol. Rev.* **92**, 1651-1697 (2012).

7. U. Proske, A. K. Wise, J. E. Gregory, The role of muscle receptors in the detection of movements. *Prog. Neurobiol.* **60**, 85-96 (2000).

8. G. G. Simoneau, J. A. Derr, J. S. Ulbrecht, M. B. Becker, P. R. Cavanagh, Diabetic sensory neuropathy effect on ankle joint movement perception. *Arch. Phys. Med. Rehabil.* **77**, 453-460 (1996).

9. R. Fitzpatrick, D. I. McCloskey, Proprioceptive, visual and vestibular thresholds for the perception of sway during standing in humans. *J. Physiol.* **478 ( Pt 1)**, 173-186 (1994).

10. K. M. Refshauge, R. C. Fitzpatrick, Perception of movement at the human ankle: effects of leg position. *J. Physiol.* **488 ( Pt 1)**, 243-248 (1995).

11. D. G. Thelen, C. Brockmiller, J. A. Ashton-Miller, A. B. Schultz, N. B. Alexander, Thresholds for sensing foot dorsi- and plantarflexion during upright stance: effects of age and velocity. *J. Gerontol. A Biol. Sci. Med. Sci.* **53**, M33-38 (1998).

12. L. Bringoux, V. Nougier, P. A. Barraud, L. Marin, C. Raphel, Contribution of somesthetic information to the perception of body orientation in the pitch dimension. *Q. J. Exp. Psychol. A* **56**, 909-923 (2003).

13. Z. J. Djajadikarta, S. C. Gandevia, J. L. Taylor, Age has no effect on ankle proprioception when movement history is controlled. *J Appl Physiol (1985)* **128**, 1365-1372 (2020).
